# Moodle, une pédagogie alternative crédible d’enseignement de la médecine en milieu tropical pour répondre aux grands nombres et aux situations de pandémie?

**DOI:** 10.48327/mtsimagazine.n1.2021.76

**Published:** 2021-03-29

**Authors:** A. Koama, S.L.C. Yaméogo, B.P. Yaméogo, M. Windsouri, A. Djiguemdé, N. Zongo, A. Aubrège

**Affiliations:** 1 Service de radiologie CHR de Koudougou, 01 BP 300, Burkina Faso; 2 Service de chirurgie, CHU de Tengandogo (CHUT), Burkina Faso; 3 Service de chirurgie, CHU Yalgado Ouédraogo (CHUYO), Burkina Faso; 4 Université Joseph Ki Zerbo, 03 BP 7021, Ouagadougou, Burkina Faso; 5 Université de Nancy, France adjikoama@gmail.com

**Keywords:** Moodle, Enseignement, Médecine, Afrique, Moodle, Teaching, Medicine, Africa

## Abstract

**Introduction:**

Moodle est une plateforme interactive d’enseignement en ligne, un véritable amphithéâtre virtuel, supprimant les barrières géographiques et d’espaces, capable d’offrir un enseignement continu même en période de guerre ou de pandémie.

**But:**

montrer la contribution possible de Moodle dans l’enseignement de la médecine en milieu tropical pour répondre aux grands nombres et aux situations de pandémie.

**Méthodologie:**

Il s’est agi d’une enquête transversale et descriptive. Elle a été menée en juin 2019 à l’Université Joseph KI-ZERBO de Ouagadougou au Burkina Faso. Elle a consisté en un questionnaire soumis aux étudiants de master 1 de médecine et à des enseignants. L’enquête portait sur les solutions que les étudiants proposeraient pour améliorer leurs conditions d’apprentissage, au rôle que Moodle pourrait jouer dans l’amélioration de leur apprentissage, ainsi qu’aux préalables à résoudre avant l’adoption de Moodle. Le taux de répondant était de 75,6 % de l’ensemble de la promotion.

**Résultats:**

Pour les étudiants Moodle est une bonne alternative à l’enseignement classique. Cependant des conditions préalables sont à remplir telles que le don d’ordinateur portable à chaque étudiant (90,3 %), la disponibilisation d’une bonne connexion à l’internet pour tous (96 %), leur formation préalable à l’utilisation de Moodle. Les étudiants ne trouvaient pas d’inconvénient pour leur évaluation en ligne via Moodle à condition qu’elle soit fiable et vérifiable en cas de besoin. Les enseignants dans 38 % souhaitaient que l’enseignement reste classique, présentiel contre 62 % qui préconisaient les nouvelles méthodes d’enseignement comme Moodle, pour peu que les conditions propices soient mises en place.

**Conclusion:**

Moodle est un e-learning crédible pouvant faciliter la gestion des grands nombres et assurer la continuité des enseignements en situation de pandémie malgré les besoins de confinement. Cependant, il doit être une technique maîtrisée, appliquée sur un terrain préparé.

## Abstract (en)

**Introduction:**

Moodle is an interactive online education platform, a true virtual amphitheater, breaking down geographical and space barriers, capable of offering continuous education even in times of war or pandemic.

**Aim:**

To show the possible contribution of Moodle in the teaching of medicine in tropical environments to respond to large numbers and pandemic situations.

**Methodology:**

It was a cross-sectional and descriptive survey. It was conducted in June 2019 at Joseph KI-ZERBO University of Ouagadougou in Burkina Faso. It consisted of a questionnaire submitted to Master 1 students of medicine and to their teachers. The survey focused on the solutions that students would propose to improve their learning conditions, the role that Moodle could play in improving their learning, as well as the prerequisites to be resolved before adopting Moodle. The respondent rate was 75.6% of the entire promotion.

**Results:**

For students Moodle is a good alternative to conventional education. However, prerequisites must be fulfilled such as the donation of computer to each student (90.3%), the availability of a good connection for all (96%), their training prior to the use of Moodle. Students found no harm in their online assessment via Moodle provided it was reliable and verifiable when needed. Teachers in 38% wanted teaching to remain traditional, face to face against 62% who advocated its abandonment in favor of new teaching methods like Moodle provided that the right conditions are in place.

**Conclusion:**

Moodle is a credible e-learning that can facilitate the management of large numbers and ensure the continuity of lessons learned in a pandemic situation despite the need for containment. However, it must be a mastered technique, applied on prepared ground.

## Introduction

L’enseignant traditionnel comprenait un expert, un détenteur et transmetteur du savoir appelé enseignant placé devant des personnes à la quête du savoir, assoiffées d’apprentissage appelées étudiants dans un endroit précis appelé classe ou amphithéâtre pendant des heures préétablies de la journée [1,2,3]. Cette conception avec le développement des moyens de communication sans être abandonnée s’est avérée présenter des contraintes évitables de lieu, d’horaires et dotée d’une conception de la relation enseignant-enseigné perfectible. Cette relation se veut d’être désormais une relation de collaboration entre un médiateur (enseignant) et un constructeur actif de savoir (enseigné) en lieu et place de la traditionnelle relation transmetteur-receveur de savoir. Les deux acteurs se trouvant le plus souvent en des endroits différents, reliés par une plateforme disponible sur internet [1,2,3,4]. C’est le e-learning [2,3,4,5]. La méthode traditionnelle d’enseigner a été bousculée cette dernière décennie par la quête perpétuelle du meilleur rapport coût/efficacité de l’enseignement partout dans le monde [2]. Le e-learning a par conséquent pris toute sa place [3, 6]. Il est largement utilisé dans les pays industrialisés qui disposent de moyens de communication adaptés. Il rend les étudiants plus créatifs en leur laissant un temps suffisant de réflexion, des heures de travail à volonté et rend d’énormes services économiques en réduisant les coûts de formation [4]. Bien qu’encore timide dans les pays en voie de développement, le e-learning est très vite apparu comme une nécessité vitale pour ces pays qui aspirent au bien-être grâce à une meilleure éducation, un meilleur enseignement [2]. L’explosion démographique a augmenté considérablement lenombre d’étudiants dans ces pays mettant à rude épreuve la capacité des amphithéâtres. Entre la volonté de former suffisamment de cadres et la capacité de les former dans de bonnes conditions, les moyens font souvent défaut. Le e-learning pourrait dans certaines conditions être une alternative pour pallier au manque de place dans les amphithéâtres et réduire les coûts de déplacement et la propagation des maladies en situation de pandémie. A l’instar des autres pays d’Afrique, au Burkina Faso, les enseignants sont de plus en plus formés à l’enseignement en ligne par l’usage de la plateforme Moodle. Moddle est une plateforme interactive, une véritable classe virtuelle, permettant d’enseigner et d’évaluer sans besoin de contact entre enseignant et enseignés [2,4]. Avant que Moodle ne rentre pleinement en action comme modèle d’enseignement à l’Université Joseph KI-ZERBO de Ouagadougou, nous avions voulu savoir ce qu’en pensent les étudiants de la faculté de médecine. C’est pourquoi nous avions entrepris cette enquête auprès de la promotion de la faculté de médecine qui comptait le plus grand effectif d’étudiants de toute l’histoire de la médecine du pays, pour savoir si Moodle était pour eux une alternative crédible en ce qui concerne l’enseignement théorique.

## MÉthodologie

Il s’est agi d’une enquête transversale et descriptive. Elle a été réalisée le 25 juin 2019 dans l’amphi B de l’Université Joseph KI-ZERBO de Ouagadougou de 15h30 à 16H30. Elle s’est intéressée aux étudiants de master 1 de médecine. Ils sont une promotion de 1035 étudiants et constituent la plus grande promotion de l’histoire de l’enseignement de la médecine du pays. La faculté de médecine est fonctionnelle de façon continue depuis 1981 et n’avait jamais enregistré une promotion de cette taille. De façon pratique, des fiches d’enquêtes préalablement préparées ont été distribuées aux étudiants présents. Avant qu’on ne leur demande de répondre aux questions qui s’y trouvaient, nous avons expliqué de façon succincte Moodle. Les explications ont concerné son principe de fonctionnement, ses objectifs, ses implications logistiques, l’engagement requis pour le corps enseignant et les étudiants, ses atouts mais aussi ses limites. La taille minimale de l’échantillon pour obtenir une représentativité au niveau de cette promotion et valider le questionnaire a été calculée selon la formule suivante: n =z^2^p (1-p)/e^2^ (z=1,96; e = 0,05 marge d’erreur acceptée; p = 0,5). En tenant compte des enquêtes similaires, un taux de non-répondants de 5 % était prévisible. Ainsi une taille minimale de 385 + 5 % (385) a été retenue, soit un nombre minimal de 405 répondants. Le jour de l’enquête, 819 étudiants étaient présents dans l’amphithéâtre. Sept cent quatre-vingt-trois (783) ont répondus au questionnaire, soit un taux de répondant de 75,6 % (783/1035). Sept cent quarante-deux (742) fiches ont été complètement remplies. Toutes ces fiches ont été incluses dans l’étude. Un prétest avait été fait avec les étudiants de master 1 de l’université de SAABA pour valider le questionnaire. Un enseignant de master 1, et 6 étudiants en médecine se sont chargés de la distribution des fiches d’enquête. Une réunion préliminaire entre enquêteurs avait été faite pour uniformiser la démarche, la durée de l’enquête, et parer aux éventuelles questions des enquêtés sans orienter ou influencer leurs réponses. La fiche de collecte contenait des questions fermées, semi-fermées et ouvertes. Nous nous sommes intéressés aux conditions de participation aux cours de 1 035 étudiants dans un amphi de 600 places règlementaires, aux solutions que les étudiants eux-mêmes proposeraient pour améliorer leurs conditions d’apprentissage, au rôle que Moodle pourrait jouer dans l’amélioration de leur apprentissage, ainsi qu’aux préalables avant l’adoption de Moodle. Un deuxième questionnaire a été également soumis à 42 enseignants ayant bénéficié déjà de la formation Moodle.

## RÉsultats

Une promotion de 1 035 étudiants prend les cours de médecine dans un amphi de 600 places réglementaires. Des chaises sont disposées dans les allées pour offrir le plus grand nombre de place possible. Les cours sont données sous forme de polycopiés appuyés par des cours théoriques. Les étudiants ont estimé à 80 % le nombre total d’étudiants présents régulièrement dans l’amphithéâtre pendant les cours. Le jour de l’enquête, ils n’étaient que 75,6 % de l’ensemble de la promotion, soit 783 étudiants dont 742 ont fourni des fiches d’enquête bien remplies. Il y avait 252 étudiants absents ce jour. Pour 61 % (453) des répondants aux questionnaires, le manque de place serait responsable des absences. Pour 100 % (742) des répondants, la solution au grand nombre réside dans la construction d’autres amphithéâtres. Seulement 36 % (267) ont estimés qu’il faut réduire le nombre d’entrée en médecine par l’instauration du numerus clausus. Ils proposent un système de double flux dans 45 % des cas. D’autres systèmes d’enseignement comme Moodle ont été souhaités par 78 % des répondants à l’enquête. Les avantages évoqués tel que la suppression des déplacements ont été résumés dans le tableau I. Pour les répondants, les inconvénients seraient l’absence de contact suffisant entre promotionnaires pendant le cours théorique dans 48 % des cas. Mais, ils ont signalé que cela pourrait se rattraper sur le terrain de stage. Pour que Moodle soit une bonne alternative, à l’enseignement classique, des conditions préalables sont à remplir telles que le don d’ordinateur portable à chaque étudiant (90,3 %), l’amélioration du système de connexion à internet dans le pays (96 %). Ces préalables ont été résumés dans le tableau II. Les étudiants ne trouvaient pas d’inconvénient pour leur évaluation en ligne à condition qu’elle soit fiable et vérifiable en cas de besoin. Un questionnaire joint en annexes a été soumis aux enseignants formés à l’utilisation de Moodle. Sur les 42 enseignants ayant accepté répondre au questionnaire, 38 % souhaitent que l’enseignement reste classique, présentiel contre 62 % qui préconisaient son abandon au profit des méthodes nouvelles d’enseignement comme Moodle. Les enseignants dans 90,5 %, ont préconisé un enseignement mixte comprenant un cours théorique via la plateforme Moodle et des travaux pratiques en présentiel avec une répartition des étudiants en petits groupes. Pour le nombre pléthorique, les enseignants ont souhaité à l’unanimité (42/42), l’instauration d’un numerus clausus prenant seulement les 200 meilleurs étudiants à la fin de la première année de médecine. En outre l’instauration d’un quota pour les étrangers permettrait d’avoir un nombre d’étudiants acceptable vu que ces derniers représentaient près de 50 % dans certaines promotions.

**Tableau I:** Répartition des étudiants en fonction des avantages de Moodle, n=742 Distribution of students by Moodle benefits, n = 742

Avantages	Nombre	Pourcentage (%)
Amphi virtuel, pas besoin de place	499	67,3

Bonne structuration des cours	606	81,7

Cours disponibles en permanence	672	90,6

Pas besoins de se déplacer	402	54,8

Éviction de la timidité	102	13,8

Horaires souples	721	97,2

Obtention rapide des résultats d’évaluation	729	98,2

Éviction des humeurs de certains enseignants	73	9,9

Éviction du mauvais comportement de certains étudiants	94	12,6

**Tableau II:** Répartition des étudiants en fonction des conditions souhaitées avant l’adoption de Moodle n=742 >Distribution of students according to the desired conditions prior to Moodle adoption n = 742

Conditions préalables	Nombre	Pourcentage (%)
Kit informatique	670	90,3

Abonnement annuel	622	90,6

Formation en informatique de base	482	65

Formation sur Moodle	742	100

Amélioration du système de connexion dans le pays	712	96

## Discussion

Le souci permanent de disponibiliser des médecins pour tous les citoyens pousse les gouvernants à encourager les meilleurs élèves à s’orienter en médecine après l’obtention du baccalauréat. L’enseignement de la médecine a ses prérogatives. La bonne transmission du savoir nécessite un enseignement théorique de qualité et un followship sur les terrains de stages pratiques. Plusieurs méthodes sont utilisées [1,2,3]. La méthode traditionnelle livre un enseignement en présentiel, réunissant dans un amphi limité en nombre de place un détenteur du savoir appelé enseignant et des étudiants en quête du savoir [1,3]. Elle nécessite un déplacement de tous les concernés [1]. Ce modèle d’enseignement même s’il semble garantir une meilleure qualité de transmission du savoir reste limité par les moyens qu’il suscite et n’est possible qu’en temps de paix [7]. La pandémie à corona virus vient nous rappeler si cela en était utile que l’espace et la liberté de se déplacer dans tous les sens ne nous seront pas toujours garantis [7]. Le confinement déclenché par cette pandémie a entraîné la fermeture de toutes les universités avec un arrêt total des cours pour les pays ne disposant pas d’autres modalités d’enseignement comme le Burkina Faso. C’est toute l’importance des e-learning [8].

Ils poursuivent un double objectif, conserver la qualité tout en réduisant de façon drastique le coût de l’enseignement. Ils garantissent aussi sa continuité même en période de guerre, de pandémie et autorisent la formation d’un plus grand nombre [9,10]. C’est ainsi que des plateformes de cours en lignes sont mise en place. Un étudiant peut alors à partir de Tokyo, Berlin, Lomé, Abidjan ou Parakou suivre des cours en ligne à Paris et obtenir des diplômes gratifiants [9,10].

Avec le galop démographique, les pays pauvres ont de plus en plus de problème à trouver des amphis de bonne taille pour recevoir les étudiants dont le nombre ne cesse d’augmenter. Dans cette lancée Moodle est une plateforme interactive, une véritable classe virtuelle, permettant d’enseigner et d’évaluer sans besoin de contact entre enseignant et enseignés [1,3]. Elle a déjà fait ses preuves, sa capacité à fournir des enseignements de qualité dans plusieurs pays [11,12]. Au Pakistan, il a été utilisé de façon mixte avec l’enseignement classique et a montré son intérêt chez les étudiants ayant une bonne connaissance de l’outil informatique [2]. Au Burkina Faso, plusieurs enseignants sont formés à son utilisation au compte de l’université Joseph KI-ZERBO de Ouagadougou. Pour ces enseignants formés ayant une bonne connaissance des avantages et des inconvénients de l’outil, 62 % prônent son utilisation dans l’enseignement de la médecine. Cependant ils ne souhaitent pas qu’il soit utilisé pour augmenter le nombre d’étudiants (100 %) mais pour améliorer la qualité de l’enseignement et garantir sa continuité en période de pandémie ou de guerre. En effet Moodle en imposant un modèle de format des cours aux enseignants harmonisera le plan des cours, et aussi le contenu [1,3]. Les étudiants auront accès aux cours à tout moment et cela va nécessairement augmenter leur volume horaire de travail [10,12]. En outre les enseignants sans contrainte de temps pourront mettre sur la plate-forme beaucoup plus d’exercices qu’en présentiel voire des courtes vidéos de démonstration [1,6]. Une bibliothèque virtuelle pourrait être crée sur la plateforme et contenir des centaines de livres utiles et les résultats des travaux scientifiques des enseignants [1,5]. Une telle plateforme accessible à tous les étudiants inscrits améliorera dans ce sens le processus d’acquisition de connaissances [1,5]. Moodle est une bonne manière d’enseigner pour les cours théoriques, cependant les enseignants proposent de ramener le nombre d’étudiants par promotion à des chiffres raisonnables, les former sur l’usage de Moodle, les outiller conséquemment avant de faire de Moodle le modèle d’enseignement prioritaire. Ils souhaitent en plus que cela soit utiliser en enseignement mixte comme au Pakistan [2]. Cette vision d’enseignement mixte, d’équipement est partagée par 96 % des étudiants. Cependant ils ne souhaitent pas la réduction du nombre d’étudiant par quelque mécanisme que ce soit (64 %).

En somme, enseignants et étudiants sont unanimes pour la place que pourra prendre Moodle dans l’enseignement de la médecine. Il permet de lever les barrières géographiques et d’élargir l’offre. Elle permet d’accommoder les contraintes personnelles, en particulier les contraintes liées à l’emploi du temps. Moodle nécessite cependant des investissements importants avant que les utilisateurs n’en tirent profit. Les auteurs présentent Moodle comme un outil déjà performant dans les pays riches, or son usage est certes devenu courant dans les universités, surtout avec la pandémie, mais la pratique a justement révélé les limites de l’enseignement à distance et le fait que Moodle, bien qu’étant un outil intéressant, devrait plutôt être utilisé en alternance ou de façon complémentaire aux enseignements en présentiel. La méthode d’enseignement traditionnelle, avec des étudiants passifs, est certes un peu dépassée, mais les outils de e-learning présentent, à côté de leurs qualités, des faiblesses qui mériteront des améliorations et une meilleure synergie entre différentes modalités pédagogiques, plutôt qu’une substitution.

## Conclusion

Moodle est un e-learning crédible. L’actualité avec la pandémie du COVID 19, ayant entraîné la fermeture des universités partout dans le monde, viens nous rappeler l’utilité et la nécessité de ces plates-forme d’enseignement à distance. Il doit être une technique maîtrisée, appliquée sur un terrain préparé. N’ayant pas besoin de regroupement des étudiants, il facilitera la gestion des grands nombres mais aussi la poursuite de l’enseignement même en situation de pandémie tout en respectant les besoins de confinement. Il parait donc urgent de réunir les conditions de son application.

## Conflits D'intérêts

Les auteurs ne déclarent aucun conflit d’intérêts.
